# How Self-tracking and the Quantified Self Promote Health and Well-being: Systematic Review

**DOI:** 10.2196/25171

**Published:** 2021-09-21

**Authors:** Shan Feng, Matti Mäntymäki, Amandeep Dhir, Hannu Salmela

**Affiliations:** 1 Department of Management and Entrepreneurship, Turku School of Economics University of Turku Turku Finland; 2 Department of Management, School of Business and Law University of Agder Kristiansand Norway

**Keywords:** self-tracking, quantified self, health, well-being, systematic literature review, literature review

## Abstract

**Background:**

Self-tracking technologies are widely used in people’s daily lives and health care. Academic research on self-tracking and the quantified self has also accumulated rapidly in recent years. Surprisingly, there is a paucity of research that reviews, classifies, and synthesizes the state of the art with respect to self-tracking and the quantified self.

**Objective:**

Our objective was to identify the state of the art of self-tracking and the quantified self in terms of health and well-being.

**Methods:**

We have undertaken a systematic literature review on self-tracking and the quantified self in promoting health and well-being. After a rigorous literature search, followed by inclusions, exclusions, and the application of article quality assessment protocols, 67 empirical studies qualified for the review.

**Results:**

Our results demonstrate that prior research has focused on 3 stakeholders with respect to self-tracking and the quantified self, namely end users, patients and people with illnesses, and health care professionals and caregivers. We used these stakeholder groups to cluster the research themes of the reviewed studies. We identified 11 research themes. There are 6 themes under the end-user cluster: user motivation and goal setting, usage and effects of self-tracking, continuance intention and long-term usage, management of personal data, rejection and discontinuance, and user characteristics. The patient and people with illnesses cluster contains three themes: usage experience of patients and people with illnesses, management of patient-generated data, and advantages and disadvantages in the clinical context. The health care professional and caregiver cluster contains two themes: collaboration among patients, health care professionals, and caregivers, and changes in the roles of patients and professionals. Moreover, we classified the future research suggestions given in the literature into 5 directions in terms of research designs and research topics. Finally, based on our reflections on the observations from the review, we suggest four future research directions: (1) users’ cognitions and emotions related to processing and interpreting the information produced by tracking devices and apps; (2) the dark side of self-tracking (eg, its adverse psychosocial consequences); (3) self-tracking as a societal phenomenon; and (4) systemic impacts of self-tracking on health care and the actors involved.

**Conclusions:**

This systematic literature review contributes to research and practice by assisting future research activities and providing practitioners with a concise overview of the state of the art of self-tracking and the quantified self.

## Introduction

Self-tracking—also referred to as the quantified self, self-monitoring, lifelogging, personal analytics, and personal informatics [1]—has become pervasive in people’s daily lives and increasingly common in health care. For example, health and fitness ranked ninth out of 34 categories on the App Store, accounting for 3.41% of all the available apps [2,3]. In Google Play, health and fitness apps ranked 13th out of 33, accounting for 3.53% of all the available apps [4]. Moreover, the App Store hosted 48,608 apps for health care purposes, and Google Play 47,140 in the third quarter during 2020 [5,6]. Although global smartwatch shipments have been estimated to exceed 100 million units in 2020 and continue to grow in 2021 [7], self-tracking tools have become available for increasing numbers of people across the globe.

Research on self-tracking and the quantified self has proliferated rapidly in recent years [8-14]. This growth calls for review studies that help enrich the knowledge base by classifying and synthesizing prior research and providing directions for future research activities [15,16]. However, dedicated review studies are scant in the most recent literature on self-tracking and the quantified self. Altogether, the extant literature features 8 review studies [17-24], of which 5 have been published in journals [17-19,22,24] and 3 in conference proceedings [20,21,23].

Moreover, some of the prior review studies have not focused exclusively on self-tracking or the quantified self. For example, Paton et al [18] focused on wearable self-tracking devices, social media platforms used by the self-tracking community, and personal health records. West et al [19] conducted a review of self-tracking in the context of patient-generated data, whereas Lentferink et al [24] identified the key components of self-tracking and persuasive eCoaching in automated healthy lifestyle interventions to improve the design of these interventions. Therefore, a review focusing only on the quantified self and self-tracking is needed.

Third, with respect to the scope, prior review studies have typically focused on a certain subset of the literature. For example, Moya et al [20] focused on the adoption and utilization of self-tracking technologies. Almalki et al [17] limited their scope to studies that applied activity theory for health self-quantification. Jiang and Cameron [22] only focused on self-monitoring in the context of chronic disease. Furthermore, most of the extant reviews covered a relatively small number of studies. The number of studies covered by the prior reviews are 43 [21], 32 [24], 28 [23], 26 [17], and 23 [19] respectively, and one of the prior reviews did not clearly mention the number of reviewed studies. Considering these limitations in terms of the scope of prior reviews and the increasing research volume on self-tracking and the quantified self, there is a need for an up-to-date and comprehensive literature review.

To address this gap in the literature, we undertook a systematic literature review (SLR) of the research on self-tracking and the quantified self in terms of health and well-being. The purpose of this study can be summarized as follows: (1) to identify high-quality contributions in the area, (2) to classify the literature based on publication channels, research methods, theoretical backgrounds, and variables used, (3) to synthesize the main research areas and research themes, and (4) to identify future research directions. The final pool of the reviewed articles comprised 67 empirical studies.

We mapped the research subjects of the reviewed articles and identified three main stakeholder groups: end users, patients and people with illnesses, and health care professionals and caregivers. For the end users, the primary usage of self-tracking is for tracking exercise, daily activity levels, and sleep. For the two other stakeholder groups (ie, patients and people with illnesses as well as health care professionals and caregivers), the primary usage of self-tracking is to support the treatment of an illness or other medical conditions. We further used these stakeholder groups for clustering the research themes of the reviewed studies. By mapping the research themes, examining the focal constructs of the prior studies, synthesizing the future research directions, and finally reflecting on the findings and suggesting new research areas, this study aids in building future research efforts [15,16].

The remainder of the paper is structured as follows. The background and related works are discussed next followed by the research methodology and details of the article selection procedure. Then, we report the results of the analysis. Finally, the implications, limitations, and some directions for future research are discussed. Multimedia Appendix 1 presents the article search strategy. Multimedia Appendix 2 contains the quality assessment criteria used for selecting the articles to be included in the review. Multimedia Appendices 3, 4, and 5 contain detailed information about the reviewed articles. Multimedia Appendix 6 presents a summary of the reviewed studies based on their foci and research theme matrices.

### Background and Related Works

#### Self-tracking and the Quantified Self

Self-tracking and the quantified self are not new labels. As early as the 1970s, wearable computers for self-tracking were used as forms of personal surveillance [25]. In 2001, a small number of media practitioners began to use newly available digital technology to track their daily life for designing web 1.0 interfaces [26]. According to Lupton [1], self-tracking “involves practices in which people knowingly and purposively collect information about themselves, which they then review and consider applying in their lives.”

As further pointed out by Lupton [1], in addition to self-tracking, there are several other terms—such as lifelogging, personal informatics, and the quantified self—used to describe the practices by which people may seek to monitor their everyday life. Lifelogging can be viewed as the practice of recording information about one’s life using digital tools. Personal informatics is a term used mostly in the academic human-computer interaction community [1]. The concept of the “quantified self,” originally coined by Wolf and Kelly in 2007, refers to “self-knowledge through numbers” [1]. They used quantitative data as a means or an embodiment of monitoring the elements of everyday life. The term can be further viewed as a collaboration between users and toolmakers [27], a cultural phenomenon involving technology [8], and an outcome of self-tracking [28]. In fact, the original intention of Wolf and Kelly was to use the term “the quantified self” to describe this digital self-tracking phenomenon [1].

#### Health and Well-being

According to the World Health Organization, health is explicitly linked with well-being, which is “a state of complete physical, mental, and social well-being and not merely the absence of disease or infirmity” [29]. With the development and diffusion of wearable, wireless communication, and cloud computing technologies, self-tracking devices can not only provide data, but also refine them into key performance indicators and produce visualizations of these data. People use self-tracking technologies to self-collect personal data including biological, physical, behavioral, or environmental information [30,31]. Moreover, according to Lupton (2016), the domain of self-tracking today also includes relationships and work productivity. In the scope of self-tracking or the quantified self, health and well-being are the main tracking domains. People who knowingly and purposively track their health and well-being information (such as heart rate, sleep, physical activity, calories, clinical symptoms, stress, and recovery) to review and modify their lives widely use self-tracking devices.

#### Related Works

Self-tracking and the quantified self have been examined in various disciplines and from various theoretical and methodological premises. For example, the computer science research field has examined wearable augmented reality systems based on walking locomotion analysis [32] and developed algorithms for monitoring sleep [33]. The relevant literature also indicates certain design considerations, such as presenting negative data in a way that does not demotivate users [34]. Communication research has discussed topics such as self-tracking as a communicative phenomenon with social media, the self, and social networks of peers [35]. Furthermore, sociological studies have discussed ways of attributing meaning to data-gathering practices in terms of the quantified self [36]. Prior literature also includes research focusing on the societal and ethical concerns regarding self-tracking [37], including the value of personalized health care. In medicine, prior studies have explored the clinical experience of self-tracking technologies in the context of chronic diseases [38,39] and use of self-tracking devices in rehabilitation [40,41]. Finally, research on information systems has examined themes such as user acceptance of self-tracking, user motivation, and goal attainment related to self-tracking and the quantified self [42-44].

To keep the scope of the study manageable, we deliberately focused on self-tracking and the quantified self, executed via devices, apps, and platforms, and excluded digital diaries and video recordings to focus solely on the role of self-tracking in health and well-being. Moreover, we focused on empirical research where the primary research subjects are humans. Thus, purely technical papers and articles focusing on products, services, and markets were excluded.

## Methods

### Research Questions

The purpose of the SLR is to determine the current state of research on self-tracking and the quantified self in the domain of health and well-being. To this end, we address 6 specific research questions (RQs) that are presented in Table 1.

**Table 1:** Research questions.

Number	Research question
RQ1^a^	How has the volume of publications on self-tracking and the quantified self in the domain of health and well-being evolved?
RQ2	What are the most important publication channels?
RQ3	What research methods have been used?
RQ4	What theoretical backgrounds and variables have been employed?
RQ5	What recurring research themes can be identified from the literature?
RQ6	What future research directions can be synthesized from the literature?

^a^RQ: research question.

### Research Design

**Figure 1:**
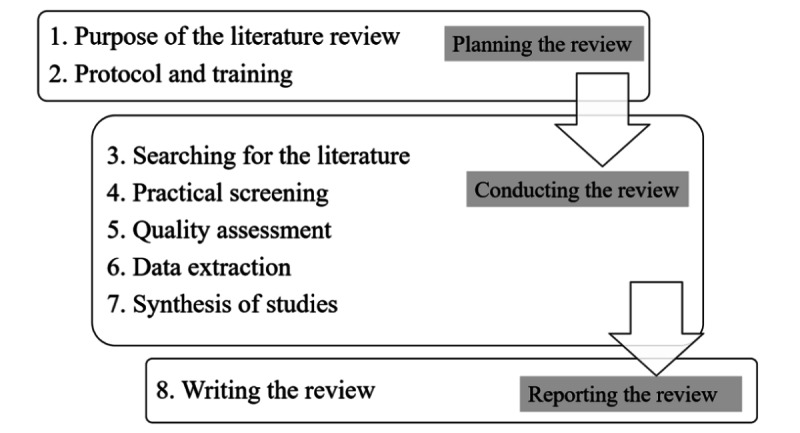
Review process.

SLRs have been developed as a specific method for identifying and synthesizing research findings [45]. They are considered particularly useful to disseminate the key findings of large and complex bodies of research. SLRs employ a transparent and rigorous approach (review protocol) to identify and synthesize all available research findings of sufficient quality concerning a specific research question or subject [46]. According to Victor [47], an SLR differs from a traditional literature review owing to its specific emphasis on the following features: (1) comprehensive coverage of the literature as far as possible; (2) paying careful attention to the quality of the included evidence; (3) taking a clear, systematic approach to data synthesis, and (4) generally following transparent and rigorous processes. In this research, we followed a well-established 8-step review protocol [48]. Figure 1 summarizes the review process.

#### Database and Search Syntax

Articles were obtained from five academic databases: Scopus, Web of Science, PubMed, Taylor & Francis, and the Association for Information Systems eLibrary. The search strategy included keywords such as “self-track*,” “self track*,” “quantified self,” “quantified-self,” “self quantif*,” and “self-quantif*.” We limited the search to journal and conference papers published in English. After conducting the literature search in the 5 databases, we eliminated duplicates. The details of the search strategy are shown in Multimedia Appendix 1.

#### Inclusion and Exclusion Criteria

Two authors (SF and MM) conducted the inclusion/exclusion procedure, prepared notes, and exchanged information throughout the process. Citation chaining was conducted to further reinforce the comprehensiveness of the article search. Textbox 1 presents the inclusion and exclusion criteria.

**Inclusion and exclusion criteria.**

**Inclusion criteria (IC)**

IC1: Studies with the main topic (title/abstract/keywords) containing self-tracking or the quantified self

IC2: Studies published in a peer-reviewed journal or scientific conference

IC3: Studies published in English

IC4: Studies with title/keywords/abstract-based screening

IC5: Studies with full-text–based screening

IC6: Studies with humans as primary empirical research subjects

IC7: Studies based on citation chaining

**Exclusion criteria (EC)**

EC1: Studies with matching title and digital object identifiers

EC2: Studies whose main topic is not self-tracking or the quantified self (digitized self-tracking and self-quantification via mobile devices, apps, and platforms; ignore diary and video recordings)

EC3: Studies where the purpose of the paper is not improving health and well-being

EC4: Studies that are not empirical studies (literature reviews, conceptual papers, technical papers, or editorials)

EC5: Studies in which the primary empirical subjects are not human, such as products, services, or markets

EC6: Studies based on quality assessment

### Quality Assessment

Quality assessment is a step in the SLR process conducted to ensure that the results of the review are suitable and impartial by identifying the articles that are not of sufficient quality to be included in the sample pool in an objective and replicable manner [48]. The quality assessment protocol incorporates the research method, structuring, implications (contributions or implications), and limitations, and ranking of the publication channel. The maximum total score from the assessment was 8.5. The criteria for quality assessment are presented in Table 2. To further ensure the high quality of the articles included in the review, we followed the approach proposed by Idri et al [49] and Behera et al [50] and set 50% of the maximum quality score as the threshold for a paper to be included in the in-depth review. Hence, 17 articles that received a total score of less than half of the maximum score (ie, 4.25) were omitted from the review. The quality score of each article can be found in Multimedia Appendix 2.

**Table 2:** Quality assessment criteria.

Criterion	Description	References
QA1^a^	The empirical study adopts a qualitative, quantitative, or mixed method approach. The possible answers are mixed method (+2), and quantitative or qualitative research (+1).	[50]
QA2	The study is a fully structured article divided into four basic sections: introduction, methods, results, and discussion. The answers are Yes (+1) and No (+0).	[51]
QA3	The study unequivocally describes the research process in sufficient detail. A quantitative study shows the questionnaire items, a qualitative one the coding and categorization process, and an experimental one the details of the experiment. The answers are Yes (+2), Partially (+1), and No (+0).	[17]
QA4	The study clearly documents the research implications (contributions or implications) and limitations. The answers are Yes (+2), Partially (+1), and No (+0).	[50]
QA5	The study was published in a reliable and recognized publication journal. Based on Journal Citation Reports (JCR: an annual publication that provides information about academic journals with impact factor data): journal in the top 25% (Q1 +1.5), in the 25%–50% group (Q2 +1), in the 50%–100% group (Q3 or Q4 +0.5), and no JCR ranking (+0). Conference ranking based on CORE (conference ranking portal): CORE A* or A (+1.5), CORE B (+1), CORE C (+0.5), and no CORE ranking (+0).	[49,52]

^a^QA: quality assessment.

### Pool of Articles Included in the Review

**Figure 2:**
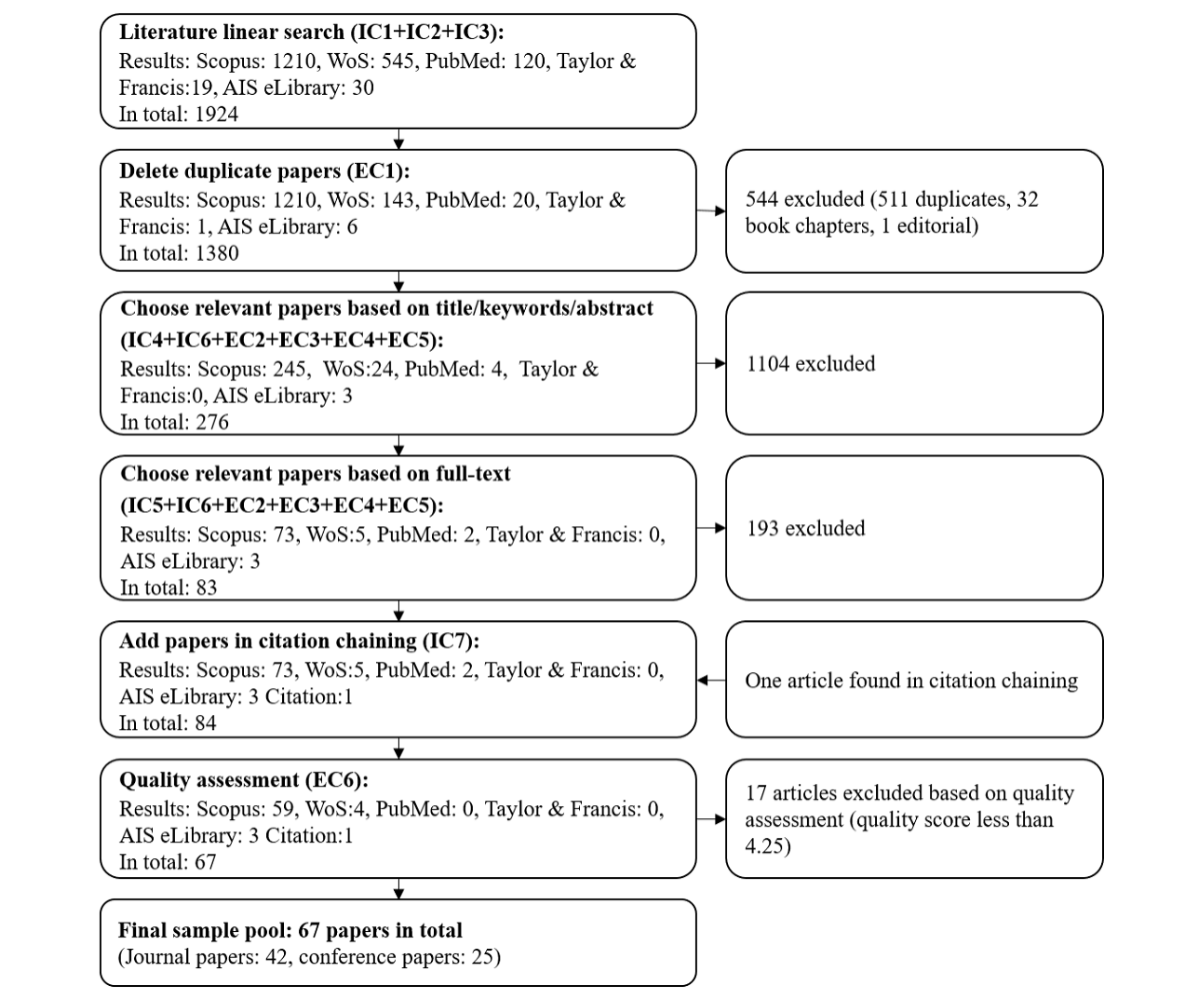
Inclusion and exclusion procedure. AIS: Association for Information Systems; IC: inclusion criterion; EC: exclusion criterion; WoS: Web of Science.

Finally, 67 articles were selected as the final sample, of which 42 were journal articles and 25 articles were published in conference proceedings. Figure 2 presents the pool of articles in each stage of the inclusion and exclusion procedure.

## Results

### RQs 1 and 2: Publications by Year and Channel

**Figure 3:**
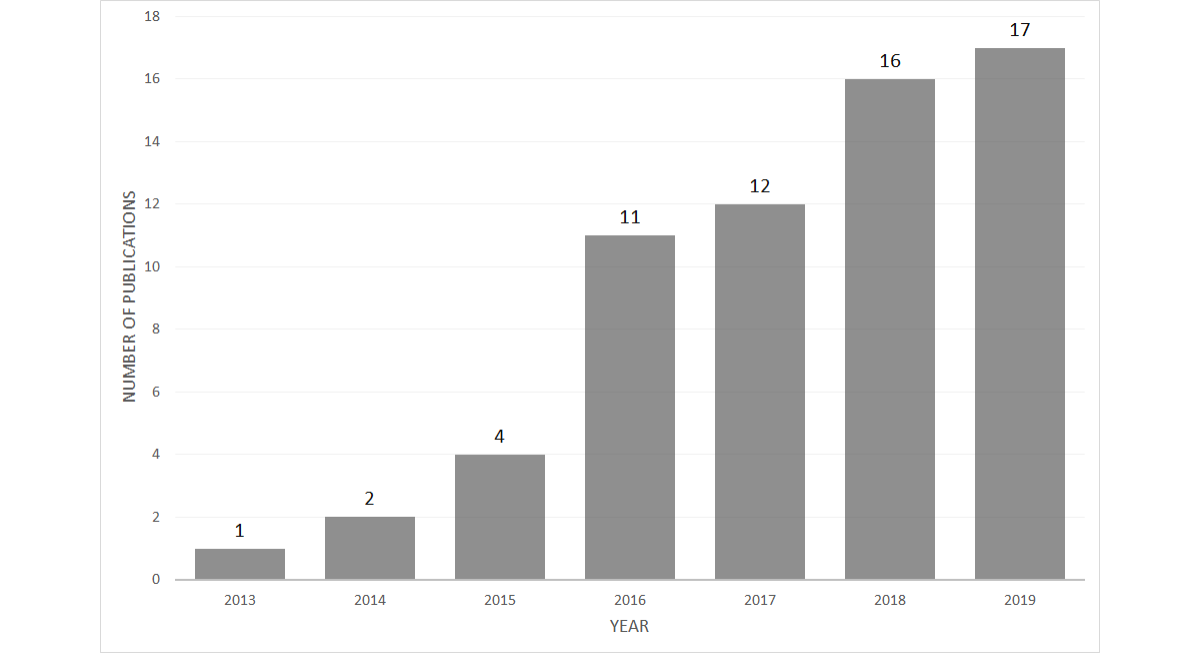
Articles included in the review by year.

In the pool of articles included in the review, the first was published in 2013. As shown in Figure 3, the number of papers published per year increased from 2013 until 2019. Table 3 presents the most popular publication channels. A considerable proportion of the conference papers appeared in information systems conferences, whereas the journal articles were published mostly in outlets focusing on health.

**Table 3:** Most common publication channels (number of publications greater than 1).

Conference/journal		Number of papers, n (%) (N=67)
**Conference**		
	Conference on Human Factors in Computing Systems	6 (8.96)
	Americas Conference on Information Systems	3 (4.48)
	Pacific Asia Conference on Information Systems	2 (2.99)
	International Conference on Information Systems	2 (2.99)
	Other conferences	12 (17.91)
	Total	25 (37.31)
**Journal**		
	Computers in Human Behavior	5 (7.46)
	JMIR mHealth and uHealth	5 (7.46)
	Journal of Medical Internet Research	4 (5.97)
	Digital Health	2 (2.99)
	Health Sociology Review	2 (2.99)
	International Journal of Human-Computer Studies	2 (2.99)
	Sociology of Health and Illness	2 (2.99)
	Telemedicine and e-Health	2 (2.99)
	Other journals	18 (26.87)
	Total	42 (62.69)

### RQ 3: Distribution of Articles by Study Method

**Figure 4:**
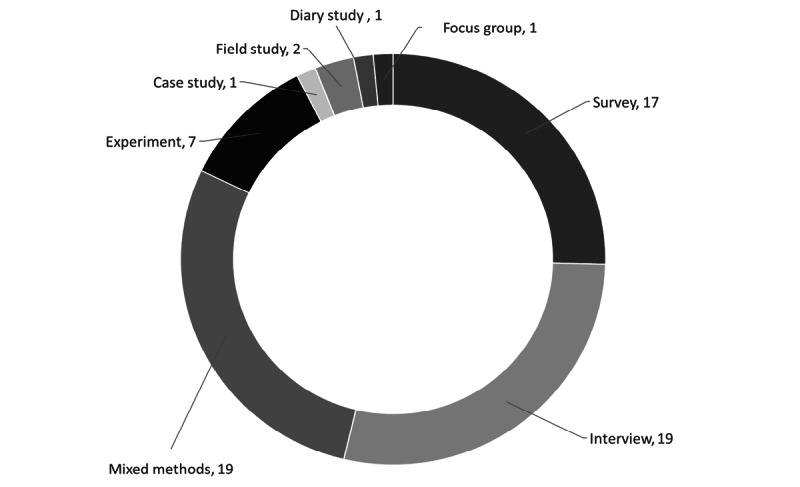
Pool of articles reviewed by research methods (total number of articles=67).

We categorized the pool of studies based on the research methods (see Figure 4). For this purpose, we examined how the empirical data were collected. The 2 most commonly used methodologies were mixed-methods research and interviews. In the mixed-methods research category, combining surveys with interviews was the most frequently used investigative approach. Most of the experimental research was conducted in a longitudinal fashion. Multimedia Appendices 3, 4, and 5 provide more information about the reviewed studies, including theories, methods, and focal constructs.

### RQ 4: Theoretical Backgrounds and Variables on Self-tracking and the Quantified Self

In our pool of 67 studies, 39 articles explicitly stated the theoretical foundation on which their studies were based. The remaining articles typically discussed prior literature but did not explicitly build on any theoretical foundation. This includes articles using the grounded theory methodology to build or contribute to theory [38,53-55]. In the aforementioned 39 articles, 18 studies used theories to develop hypotheses and research models to be tested with quantitative methods, whereas in 21 studies, the primary role of the theory was to support qualitative research.

Among the 18 quantitative studies with explicitly stated theoretical backgrounds, the most widely used theoretical lenses were the technology acceptance model (TAM) and self-determination theory. Understandably, these theoretical backgrounds were common among studies focusing on self-tracking behaviors, motivations, goals, and use continuance. Table 4 provides information about the theoretical backgrounds and dependent variables of the studies where the theoretical foundation was explicitly stated. Multimedia Appendices 3, 4, and 5 provide more details about the reviewed articles [56-68].

**Table 4:** Theoretical backgrounds and dependent variables.

Dependent variable categories		Theory/theories	References
**Self-tracking behavior/technology usage**			
	Habitual web-based fitness community use	Self-determination theory, technology acceptance model	[68]
	Number of activities	Social cognitive theory	[56]
	Behavior (activity index + sleep index + food index)	Health information technology acceptance model	[57]
	Behavior (compliance of self-tracking behavior)	Health information technology acceptance model	[58]
	Behavioral intention to use (wearable devices in the workplace)	Self-determination theory, technology acceptance model	[59]
**Motivation/goal to use self-tracking technologies**			
	Classes of motivational designs (gamification, social networking, and self-quantification)	Self-determination theory, social comparison theory, goal-setting theory	[60]
	Self-tracking motivation fulfillment	Gimpel five-factor framework of self-tracking motivations	[11]
**Continuance intention of self-tracking**			
	Continuance intention (of using smartwatches)	Net valence framework	[61]
	Continuance intention (of using quantified-self technology)	Motivational affordance theory, technology continuance theory	[62]
	Continuance intention (of the quantified self)	Trans-theoretical model, expectation-confirmation theory	[63]
**Cognitive dissonance when using self-tracking technologies**			
	Awareness of two inconsistent cognitions, self-tracking usage, and cognitive dissonance	Cognitive dissonance theory	[64-66]
**Self-disclosure of personal information**			
	Self-disclosure (personal information in self-tracking apps)	Stage-based model, five-factor model	[28]
**Dependency effect** **on** **wearing activity trackers**			
	Dependency effect (on wearing activity trackers)	Self-determination theory	[10]
**Health consciousness, physical and psychological well-being**			
	Health consciousness, physical health, and psychological well-being	PERMA (positive emotion, engagement, relationships, meaning, accomplishment) model	[67]
**Sharing health-tracking records**			
	Sharing health-tracking records	Health belief model	[27]

In addition to the dependent variables presented in Table 4, we also investigated the independent, mediating, and moderating variables employed in the reviewed studies. Multimedia Appendices 3, 4, and 5 provide more details about the reviewed studies. As depicted in Table 4, the most common dependent variables were self-tracking behavior and device usage, followed by motivation and goal for using self-tracking technologies, and continuance intention of self-tracking. Independent variables in the reviewed studies were normally extracted from theories; for example, perceived ease of use and usefulness were extracted from TAM [57,59,68], 3 strategies for reducing cognitive dissonance from the cognitive dissonance theory [64-66], 5 personality traits from the big-five personality domains [28], and 5-factor motivation (self-entertainment, self-association, self-design, self-discipline, and self-healing) from the Gimpel five-factor framework of self-tracking motivations [11]. Some studies combined theory with self-tracking practices and classified independent variables into more specific categories; for example, Chuah [61] divided perceived benefits into four perspectives, including utilitarian, hedonic, social, and symbolic, and divided perceived risks into privacy-related risks and physical risks. Suh [62] specifically subdivided motivation into hedonic, utilitarian, and eudaemonic (refers to self-fulfillment and self-improvement) motivations. In addition, Hamari [60] specifically described three independent variables: goal focus (outcome and process), goal orientation (proving, avoidance, and mastery), and goal attributes (difficulty and specificity).

In addition to the aforementioned dependent variables, the reviewed literature has covered various other variables. For example, perceived empowerment and personalization had mediating effects between self-tracking technology and advice compliance [69]. Intrinsic motivation was identified a mediator between need for cognitive closure and the dependency effect on self-tracking technologies [10]. When using smartwatches, inspiration and well-being played a mediating role between the perceived benefits and risks, and continuance intention [61]. Perceived threat, perceived usefulness, perceived ease of use mediated consumers’ health statuses, beliefs and concerns, subjective norms, and self-efficacy to attitude [57]. Finally, prior research has also explored the moderating effects of demographics such as gender, education, income [70], and BMI [69].

### RQ 5: Themes in the Existing Literature

To structure the literature analysis, we developed a classification scheme. To this end, we applied the concept matrix approach presented by Webster and Watson [71]. A concept matrix represents a logical method of defining a set of “concepts” according to which the reviewed articles can be classified.

We first identified the focus of each reviewed article based on its title, abstract, research question/objective, and results. Thereafter, we classified the articles based on the subjects of the empirical research. This led to the formation of three stakeholder groups, namely end users, patients and people with illnesses, and health care professionals and caregivers. The studies adopting the end-user perspective focused on self-tracking related to personal health, well-being, fitness, or sleep. The second, (ie, the patients’ perspectives) focused on self-tracking use usage cases related to treatment of illnesses. The third, (ie, the health care professional and caregiver perspectives) focused on treatment of illnesses and medical conditions, but the primary users are the patients’ doctors, nurses, or family members. Consequently, we used the perspectives of these stakeholder groups to establish a concept matrix. Finally, we classified the studies into 11 themes that will be discussed in the following section. Multimedia Appendix 6 presents a summary of the reviewed studies based on their foci and research theme matrices.‏

### Stakeholder Group 1: End Users

#### (1) User Motivation and Goal Setting

With respect to the studies that adopted the end users’ perspectives, user motivation constitutes an important stream of research. Most of the research examined the motivation for self-tracking usage [10-12,62,72]. In their research, Attig and Franke [10] divided the motivation for tracking physical activity into intrinsic and extrinsic motivations. The results revealed that people with strong extrinsic motivation may be highly dependent on the use of the tracking devices. Suh [62] classified motivation into hedonic, utilitarian, and eudaemonic. The results illustrated that unlike hedonic motivation, utilitarian and eudaemonic motivations positively impact continuance intention use of self-tracking technologies. Further, Pingo and Narayan [12] suggested that users may experience gratification when they can use tracking devices to control their health status. To clarify the reason for the usage of tracking devices, Gimpel et al [44] employed a survey to develop a five-factor framework of self-tracking motivations. The factors were self-entertainment, self-association, self-design, self-discipline, and self-healing. Gimpel et al [11] also illustrated that only the motivation for self-entertainment increases the wearable self-tracking device usage, which in turn influences the fulfillment of the self-entertainment motivation. Baudier et al [59] also emphasized that self-entertainment and self-design have a positive effect on the behavioral intention to use the health care Internet of Things in the workplace.

In prior research, goal setting and motivation always appeared at the same time. Gordon et al [13] found that users of weight loss–tracking apps preferred to choose achieving goals as their motivation. Self-monitoring motivation and attitude toward weight loss goals are vital for predicting goal achievement. For fitness tracking apps and exercise encouragement apps, Rockmann and Gewald [14], and Hamari et al [60] noted individual differences and found that different motivational affordances, such as self-quantification, gamification, and social networking capabilities, help users achieve their goals.

#### (2) Usage and Effects of Self-tracking

The second cluster under the end users’ perspective focused on the different uses of self-tracking and the outcomes of usage. With respect to the different uses and use cases, prior research has explored the use of self-tracking for physical activities [8,9,35,57,58,73-77], sleep [57,58,74,78], diet [57,58], fertility [55], caloric intake [79], and alcohol-harm reduction [80].

Prior literature also provides classifications of users and self-tracking tools. Makkonen et al [81] identified 4 distinct consumer segments of self-tracking based on their technology adoption patterns: pro-trackers, semitrackers, interested trackers, and nontrackers. In the context of app usage for alcohol consumption reduction, Milward et al [80] identified three types of users: the trackers, cut-downers, and non-committers. Spotswood et al [9] explored the role of self-tracking in supporting healthy behavior and found three mechanisms for teleoaffective shaping: labeling, rewarding, and materializing effort. Lyall and Robards [82] identified three roles of self-tracking devices for the user, namely as a tool, toy, and tutor.

Moreover, previous research has explored the outcomes of self-tracking. Shin and Biocca [63] noted that health consciousness is the most significant factor for staying healthy. Stiglbauer, Weber, and Batinic [67] proved that wearing a fitness tracking device can make users more conscious regarding their physical health but did not have a significant effect on their mental health. Furthermore, according to Ravichandran [78], sleep-tracking devices can help users better understand and improve their overall sleep habits. Rönkkö [74] proposed that certain design features such as graphical feedback, information sharing, and social communities in self-tracking devices may be particularly important in contributing toward positive lifestyle changes, with the most important factor being the presence of personal long-term goals.

#### (3) Continuance Intention and Long-term Usage

The third cluster of studies from the end users’ perspectives focused on sustained usage of self-tracking and the quantified self. For example, Chuah [61] found that the perceived benefits (utilitarian, hedonic, social, and symbolic) indirectly affect users’ continuance intentions regarding smartwatch use through inspiration and well-being. Shin and Biocca [63] investigated the relative effects of hedonic and utilitarian motives as determinants of confirmation, satisfaction, and continuance intention regarding wearable devices. In contrast, Suh [62] observed that hedonic motivation has a negative influence on the continuance intention when using self-tracking devices, but utilitarian and eudaemonic motivations have a positive effect. As for web-based fitness community usage, Stragier et al [68] proposed that self-regulatory and social motives directly predict sustained web-based fitness community usage. Rockmann et al [83] also theorized an emotional “carry-over effect” in activity continuance decisions on activity tracking.

With respect to the long-term use of self-tracking and the quantified self, Hardey [84] employed a mixed-methods approach and found that visualization and a long-term healthy state motivate and support long-term tracking. In addition, Meyer et al [85] built 5 use cases for personal health devices that can be used as a long- and short-term comparison list. The five use cases are supporting health behavior, improved self-understanding, identification of trends and relations, decision-making, and data collection for future use.

#### (4) Management of Personal Data

The fourth cluster of studies from the end users’ perspectives emphasized managing personal data. This comprises two subtopics: coping with personal data and privacy concerns. Feng et al [86] showed that people preferred to use health or fitness apps as their personal health information management tools, and the most popular app was Fitbit. When reflecting on the personal data stored on self-tracking devices, Maltseva and Lutz [28] proposed that individuals who habitually use self-tracking apps and devices are more likely to self-disclose their personal data. When self-tracking devices are to social networks, the proportion of lower-performing friends may positively influence users’ physical activities [56]. Moreover, Yli-Kauhaluoma and Pantzar [87] examined the gap between individual experiences and self-tracking data. They found that individuals always feel upset and confused when comparing invisible or inaccurate personal health data with their daily experiences and may eventually refuse to use self-tracking devices. In another words, individuals may experience an emotional reaction, which can then stop changing their behavior [43].

Literature has also documented problems related to tracking, managing, visualizing, and using personal data [88]. Privacy issues are obviously a central concern related to sharing self-tracking data. Chen et al [89] examined the sharing of health data among college students. Their results implied that users are generally willing to share personal health data for research purposes, and the reasons for not sharing are related to privacy concerns. Gui et al [53] pointed out that when fitness devices are connected to social networks, users are encouraged to share fitness data. However, the challenges of balancing awareness and privacy issues were still prevalent. To solve this problem, Zimmer [90] employed the communication privacy management theory to better understand the privacy boundaries related to personal fitness information and found that the advantages outweigh the disadvantages.

#### (5) Rejection and Discontinuance

The fifth cluster of studies from the end users’ perspectives relates to rejection and discontinuance. In their study of experiences during the implementation of self-tracking technology, Kari et al [42] found that the experience during the initial phase of implementation will influence the decision on adoption or rejection. Moreover, previous studies have found that the abandonment of wearable fitness tracking devices can be owing to a loss of motivation, low acceptance levels for such devices, or inaccuracy and uselessness [91]. Harrison et al [92] cited tracking accuracy and device aesthetics as barriers to activity tracking. Esmonde [93] identified the following 4 strategies of resistance used by people to datafication in fitness tracking practices: excessive labeling of some forms of data rather than tracking everything, choosing not to track every day, acknowledging that they cannot be perfect and track without interruption like a machine, and accepting that people’s feelings are more important than data.

#### (6) User Characteristics

The sixth subset of studies under the end users’ perspectives focused on user characteristics. McKinney et al [94] showed that in self-tracking of food and activity levels, high health literacy supports health goals, which comprises proper understanding of a record and how to use data to support a health goal and the awareness of privacy and ownership. The digital divide may also influence the adoption of self-tracking and the quantified self [95]. According to Régnier and Chauvel, better-off individuals use self-tracking more than the socially disadvantaged [95]. In addition, Baumgart conducted a series of studies to investigate the interaction of self-tracking with users’ cognitions, behaviors, and emotions [64-66].

### Stakeholder Group 2: Patients and People With Illnesses

#### (1) Usage Experience of Patients and People With Illnesses

In the clinical context, patients, and doctors need to track health records for symptoms or contemporary sensor data. The feasibility and acceptability of using tracking technology may differ from that of healthy individuals. Beukenhorst et al [96] focused on the feasibility and acceptability of smartwatches in patients with knee osteoarthritis; people expressed enthusiasm for self-tracking of health data, but there were barriers to full engagement, such as limited battery lives, technical issues, and unfulfilled expectations. Kim et al [54] conducted a food logger applicability test on patients, and their results demonstrated a high adherence rate. However, wrong and unreasonable usage of weight loss apps may contribute to and exacerbate eating disorders [97]. Self-tracking also demonstrated potential in managing chronic diseases and rehabilitation. Goal-directed self-tracking can help people be well prepared in all stages and support chronic condition management [98]. Mishra et al [99] researched how tracking apps and technologies helped patients cope with Parkinson disease. Furthermore, Vogel et al [40] provided evidence for the positive effects of self-tracking technology on a patient’s cardiovascular system.

#### (2) Management of Patient-Generated Data

Patient-generated data for clinical purposes have also attracted research attention. Ancker et al [39] examined personal data tracking for people with multiple chronic conditions, revealing that health-tracking data may come at an emotional cost, such as depression and anxiety, which could ultimately lead to low adoption levels for consumer health information technology. Ivanov et al [27] provided insights into the influencing factors for sharing health-tracking data. They found that health motivation, the severity of the health problem or condition, and age positively influenced patients to share data with professors. People who had a certain perceived health status preferred to share data with an acquaintance.

#### (3) Advantages and Disadvantages of Self-tracking in the Clinical Context

Literature contains specialized research on the advantages and disadvantages of self-tracking in the clinical context. With the development of tracking technology, the clinical context now provides more agency, control, and information for patients, which can help them establish a relationship with professionals [100]. Piras and Miele [38] supported this idea, stating that self-tracking mediates the patient–doctor relationship.

As for the disadvantages of self-tracking, collaborations between patients and professionals involving personal data can easily cause misunderstandings regarding the patient-generated data usage, duration, and type of tracked data. There are also patient privacy issues to address [101]. In terms of special disorders, such as Parkinson disease, Riggare et al [102] indicated that self-tracking gives people a deeper understanding of their motor or nonmotor symptoms and contributes to decision-making regarding their self-care. However, the tracking workload is heavy. It is difficult to know what and how to track, and it is also difficult to ignore the risks of obsessive tracking. Therefore, it is necessary to find a proper balance between burdens and benefits [102].

### Stakeholder Group 3: Health Care Professionals and Caregivers

#### (1) Collaboration Among Patients, Health Care Professionals, and Caregivers

Self-tracking technology provides an easier way to collect patients’ health information. Health care professionals are also willing to accept self-tracking data to assess the health status of patients [39]. Therefore, patient-provider collaborations with respect to patient-generated data became one of the research topics for self-tracking usage in clinical setting. In a previous study, Chung et al [101] used a stage-based model of personal informatics and the theory of boundary negotiating artifacts to explain misunderstandings and privacy concerns in the collaboration stage. Prior research has also demonstrated that tracking apps and technologies helped patients cope with Parkinson disease. Based on the tracking data, health care partners who are friends and family members can significantly help Parkinson patients adopt positive strategies [99].

#### (2) Changes in the Roles of Patients and Professionals

Tracking technology has also shifted the roles of patients and doctors. Owing to these technological changes, patients become health managers, and doctors are health organizers [103]. Schroeder et al [98] showed that goal-directed self-tracking can help people in setting goals, preparing knowledge, and contributing to the patient-doctor collaboration. Piras and Miele [38] supported this idea, stating that patients can negotiate a satisfactory relationship with their health care providers when using self-tracking. However, there is still a gap between users, experts, and self-tracking technologies [78]. To gain a professional view of self-tracking in clinical usage, prior research has collected data from doctors, such as in the study by Gabriels and Moerenhout [103]. They conducted an interview study to explore how medical doctors evaluate self-tracking methods and the changes after using those methods. Gabriels and Moerenhout found that regarding self-care, it is important to emphasize the contextual facets of self-tracking and the involvement of the health care professionals [103].

### RQ 6: Directions for Future Research Identified From the Literature

Following our analysis of the themes in the extant literature on self-tracking and the quantified self, we move on to describing future research directions presented in the reviewed literature. Based on the analyses and conclusions, we propose 2 perspectives with 5 main future research directions from the reviewed literature: 2 from the perspective of research design, and 3 from the perspective of research topics (see Table 5).

**Table 5:** Directions for future research.

Perspective	Future research direction	Description
Research design	Employment of longitudinal research designs	Investigate the influence variables corresponding to different usage stages; changes in the evolution of an individual’s acceptance level and self-disclosure over time [28,59,91,99].
Research design	User modalities: regular users, intermittent users, nonusers, and former users	Nonuser group: nonusers and their goal orientations and perceptions about the affordances [14,102] Former users: comparative studies with short-term and long-term users to identify barriers; people who effectively quit using web-based fitness communities and wearables [53,68] Intermittent users, nonusers, and former users [95]
Research topics	Issues related to data sharing and privacy	Data sharing: active sharing and comparing of digital activity data; employees’ attitudes about sharing data; browsing others’ tracking data and sharing one’s own tracking data; means of sharing health-tracking records [8,27,56,104] Privacy and security: personal data privacy and security challenges; privacy awareness; perceived risks around data privacy for employees [27,59,105]
Research topics	Psychological and behavioral aspects of self-tracking	Dependent variables: willingness to make in-app purchases; personal health information management; underlying motivations; decisions of consumers to adopt a self‐tracking technology [61,81,86] Independent variables: intrinsic and extrinsic motivation, health literacy, duration of self-tracking, number of devices, level of analysis, and demographic characteristics [10,43,55] Moderator variables: individual characteristics and personality types [60]
Research topics	Self-tracking in clinical use	Health-tracking data and patient–doctor relationship: whether and how the data are integrated into the patient–doctor relationship; patient–provider interactions with self-tracking data [101,103] Patients’ and doctors’ attitudes toward self-tracking: exploring doctors’ communication needs and perspectives and patients’ experiences [99,103]

### Research Designs Suggested in the Literature

#### (1) Employment of Longitudinal Research Designs

Changing habits typically takes time, and changes in people’s health often occur over time. Moreover, in different stages of usage, people may have different feelings or intentions, and engagement with their health data may change over time [106]. Prior research has extensively underscored the importance of this evolution over time and that of longitudinal research [28,59,91,99]. A longitudinal research design may also provide a better understanding of the causalities by observing and explaining changes over time [28,91]. Therefore, future studies should pay close attention to the evolution of the aforementioned aspects over time. A series of interesting topics were mentioned in prior research. First, the evolution of consumers’ acceptance level could be used to discover users’ attitudes and identify the barriers for continued usage [59]. Second, in clinical settings, changes in the roles of caregivers (friends and family members who manage the patients’ diseases) in helping patients’ self-tracking should be identified via longitudinal research [99].

#### (2) User Modalities: Regular Users, Intermittent Users, Nonusers, and Former Users

As a second research direction, prior research has identified the need to shift focus from the mainstream users of self-tracking toward an increased emphasis on the modalities of use, namely intermittent users, nonusers, or prior users of self-tracking [14,53,68,95,102]. In extant literature, intermittent users comprise those who use self-tracking and self-quantification but not on a regular basis [95,99]. On the other hand, nonusers are those who are reluctant to adopt self-tracking technologies in all social milieus [95]. Former users are those who have used self-tracking or self-quantification in the past but have quit [68,95]. Focusing on the non-mainstream groups to identify and reduce the burden of self-tracking [102] as well as discovering ways to attract new users [14] are topics worthy of future research.

### Research Topics Suggested in the Literature

#### (1) Issues Related to Data Sharing and Privacy

Issues related to sharing data generated by self-tracking, data privacy, and security have frequently been proposed as areas for future research [8,27,56,59,104,105]. Different experiences and concerns may promote different attitudes toward sharing personal data [104]. For example, Zhou et al [56] indicated that sharing self-tracking data may enable social comparisons. Furthermore, multiplatform and cross-platform data sharing has become a common phenomenon, which has consequently created challenges in terms of data privacy and security [27,105]. The tracking process of data collection, analysis, and storage also raises ethical issues [59].

#### (2) Psychological and Behavioral Aspects of Self-tracking

Psychological and behavioral research encompasses themes such as users’ behaviors, attitudes, intentions, acceptance of technologies, and their respective motivations. The literature on motivation for personal health information management can provide new ideas to improve research design [86]. Existing research shows that future research will benefit from exploring the antecedents of the adoption decisions from a microlevel perspective [81]. Moreover, as the freemium business model is extremely popular among various web-based services and software firms [107,108], existing research highlights that future research could examine consumers’ willingness to make in-app purchases in the context of self-tracking and the quantified self [61].

In terms of independent variables, the subdivisions of intrinsic and extrinsic motivations should be considered in the research on usage [10]. In addition, the demographic characteristics, such as the educational level, socioeconomic status, and cultural background, could be examined as predictors of self-tracking usage [55]. Hamari et al [60] recommend that individual characteristics and personality types can be regarded as moderating variables of goal setting and the perception of motivational design.

#### (3) Self-tracking in Clinical Use

With respect to the clinical apps of self-tracking, prior research has highlighted 2 specific areas for future research. The first direction involves health-tracking data and the patient-doctor relationship [38,100,101]. For example, future studies could explore the patient-doctor interaction with self-tracking in different clinical settings [101] and integrate the health-tracking data in the patient-doctor relationship [103]. Second, patients’ and doctors’ attitudes toward self-tracking also play important roles in clinical research. Patient usage experiments and the feasibility and acceptability of self-tracking should be investigated in the future [103]. As pointed out by Mishra et al [99], self-tracking can help patients communicate with clinicians. Thus, understanding clinicians’ communication needs and attitudes toward self-tracking represents another viable future research area.

## Discussion

### Key Findings

The purpose of this study was to identify the state of the art in self-tracking and the quantified self in health and well-being. To this end, we conducted an SLR of 67 articles, comprising 42 journal articles and 25 conference papers. We have presented the development of the number of publications over time. We also identified the key academic outlets that published research on self-tracking and the quantified self, and the most prevalent research methods and theoretical foundations.

Our results demonstrate that the outputs provided by self-tracking can be used by various stakeholders. By classifying the stakeholders into end users, patients and people with illnesses, and health care professionals and caregivers, and investigating the focal themes of prior research across these 3 groups, our review provides a structured view of the extant body of knowledge. Moreover, we have classified the future research directions provided in the reviewed studies into two categories: suggestions focusing on research designs and suggestions focusing on research topics. Against this backdrop, we highlight three main findings related to the research subjects, research designs, and the role of theory stemming from the reviewed literature.

First, with respect to the stakeholders being considered as the research subjects, our results demonstrate that of the 67 studies that qualified for the review, the majority (54) focused on end users who use self-tracking to obtain feedback on their sport and fitness activities, daily activity levels, and sleep. Although self-tracking technologies are increasingly invading various clinical settings, we claim that adopting a multi-stakeholder perspective to a greater extent could be a beneficial avenue to advance research in this area.

Second, with respect to research design, considering that in many use cases reaping the potential benefits of the self-tracking requires sustained engagement in the process and use of these technologies, it is not surprising that self-tracking continuance is a key focal area of the reviewed literature. The temporal perspective has also been incorporated in the research design as 21 of the 67 reviewed studies feature longitudinal research. This observation also echoes the notion that employment of longitudinal research methods is one of the key future research directions suggested in the reviewed literature.

Third, with respect to the role of theory, 28 out of the 67 reviewed studies did not explicate or build on any theoretical foundation. In light of this observation, there is still scope for more theory-oriented research to reinforce the theoretical and conceptual foundations and enrich the knowledge base on self-tracking and the quantified self.

### Limitations

The results and implications of this review should be evaluated in the light of its limitations. There are 2 main limitations to this review that must be acknowledged. First, the scope of the review is limited by the keywords used in the article search. We specifically employed only “self-tracking” and “quantified self” as the search terms. Consequently, superordinate and subordinate words were not included in the literature search. Second, to keep the scope of the review manageable, we focused solely on empirical articles. Therefore, future research could cover nonempirical research and the superordinate and subordinate keywords of self-tracking and the quantified self.

### Future Research Directions

By presenting and summarizing the key studies in the existing body of literature in a systematic fashion, this review will assist future research activities and thus accelerate the development of the relevant research areas. To this end, our review has outlined the theories and methods used in the extant literature. Moreover, we constructed a research theme matrix from three stakeholders’ perspectives and identified 5 main future research directions provided in the reviewed literature. Beyond these contributions to research and practice, we delineate 4 primary future research directions based on our observations and reflections of the current review and provide our views on the shortcomings and limitations of the extant literature that future studies could address.

First, surprisingly few studies in our review have focused on investigating how people perceive and interpret the information produced by tracking devices [9,13,56,83]. Although studies have examined the behavioral outcomes of self-tracking, the human information processing aspects seem to have received less attention. There are two groups of factors that largely determine how users perceive information from self-tracking, namely their cognitive processes and the information along with the way the information is being provided to the users. Thus, we suggest that future research should determine how people make sense of the information produced by self-tracking to ensure that this information fits the cognitive style and knowledge structure of the users.

Second, our review revealed a significant paucity of empirical research examining the potentially adverse psychological consequences of self-tracking. This is notable considering that there is a well-established body of literature discussing self-tracking and the quantified self from a critical standpoint [109] and a stream of literature scrutinizing the so-called dark side of information technology (IT) [110,111]. Prior research in this area has classified the dark side of IT into five categories: IT-usage-related stress, work overload, interruptions, addiction, and misuse [112]. It is plausible to assume that these phenomena may also occur in relation to self-tracking and self-quantification devices. Thus, future research could, for example, examine whether the use of self-tracking and quantified-self technologies may induce health-related obsessive-compulsive thoughts and behaviors, particularly over time. Moreover, future research could examine the role of technology addiction in the continued use of self-tracking technologies.

Third, pertaining to the level of analysis, most of the reviewed studies have examined self-tracking and the quantified self essentially as individual-level phenomena. However, as argued by Lupton [113] and Sharon [37], the rise of self-tracking and the emergence of a market for health-related products and services can be related to a societal shift toward neoliberal thinking. Neoliberalism is generally associated with policies of economic liberalization. Lupton claims that individuals who use self-tracking technology “have readily adopted the subject of the responsible, entrepreneurial citizen as it is privileged in neoliberal governmentality in seeking to take action to achieve healthy and fit embodiments and engaging in self-governance” [113]. Therefore, the individual is increasingly emphasized in the societal discourse. Against this backdrop, future studies could investigate the group- and community-level implications of self-tracking and quantified self.

Fourth, related to the stakeholder perspective adopted in this study, self-tracking has the potential to reinforce people’s sense of agency in terms of understanding their health. This in turn can alter the roles of various actors and the power balance among these actors in the health care system. Hence, future studies could focus on the systemic effects of self-tracking from a societal perspective.

## Appendix

Multimedia Appendix 1 Article search strategy.

Multimedia Appendix 2 Quality assessment.

Multimedia Appendix 3 Details of the reviewed literature (quantitative research).

Multimedia Appendix 4 Details of the reviewed literature (qualitative research).

Multimedia Appendix 5 Details of the reviewed literature (mixed methods research).

Multimedia Appendix 6 Study foci and research theme matrices.

## Abbreviations

IT: information technology
RQs: research questions
SLR: systematic literature review
TAM: technology acceptance model
